# Compound CAR T-cells as a double-pronged approach for treating acute myeloid leukemia

**DOI:** 10.1038/s41375-018-0075-3

**Published:** 2018-02-25

**Authors:** Jessica C. Petrov, Masayuki Wada, Kevin G. Pinz, Lulu E. Yan, Kevin H. Chen, Xiao Shuai, Hua Liu, Xi Chen, Lai-Han Leung, Huda Salman, Nabil Hagag, Fang Liu, Xun Jiang, Yupo Ma

**Affiliations:** 1 iCell Gene Therapeutics LLC Research & Development Division Long Island High Technology Incubator, 25 Health Science Drive, Stony Brook, NY 11790 USA; 2 Department of Hematology West China Hospital, Sichuan University, Chengdu, P.R. China; 3 Department of Pathology, Stony Brook Medicine, Stony Brook, NY 11794 USA; 4 Macau Institute for Applied Research in Medicine and Health, Macau University of Science and Technology, Macau, China; 5 Department of Internal Medicine Stony Brook Medicine, Stony Brook University Medical Center, Stony Brook, NY 11794 USA; 6 Department of Hematology, Chengdu Military General Hospital, Chengdu, Sichuan P.R. China

## Abstract

Acute myeloid leukemia (AML) bears heterogeneous cells that can consequently offset killing by single-CAR-based therapy, which results in disease relapse. Leukemic stem cells (LSCs) associated with CD123 expression comprise a rare population that also plays an important role in disease progression and relapse. Here, we report on the robust anti-tumor activity of a compound CAR (cCAR) T-cell possessing discrete scFv domains targeting two different AML antigens, CD123, and CD33, simultaneously. We determined that the resulting cCAR T-cells possessed consistent, potent, and directed cytotoxicity against each target antigen population. Using four leukemia mouse models, we found superior in vivo survival after cCAR treatment. We also designed an alemtuzumab safety-switch that allowed for rapid cCAR therapy termination in vivo. These findings indicate that targeting both CD123 and CD33 on AML cells may be an effective strategy for eliminating both AML bulk disease and LSCs, and potentially prevent relapse due to antigen escape or LSC persistence.

## Introduction

AML is a hematological disease characterized by the malignant transformation and hyperproliferation of immature myeloid cells, which replace normal bone marrow cells. Current chemotherapy regimens that combine cytarabines with anthracyclines successfully treat few patients and even fewer with relapsed and/or refractory AML [1–3]. Allogeneic hematopoietic stem cell transplantation (HSCT) remains the only viable treatment option for AML, and only a limited number of patients qualify [4]. Moreover, 50–70% of patients relapse after chemotherapy and HSCT, with the 5-year survival rate at a dismal 27%. Considering the shortcomings of current AML therapy and the stagnation of treatment advances in the past few decades, new therapies are desperately needed.

CAR T-cell immunotherapy is a new and powerful therapy that has already shown utility as a curative treatment for malignant hematological diseases, most notably B-cell lymphomas and plasma cell malignancies through targeting CD19 and BCMA, respectively [5, 6]. However, substantial relapse is seen in patients one year after CAR therapy. Therefore, a single target for CAR-based treatment may not be sufficient to prevent disease relapse. It follows that compound targeting of more than one antigen represents a critical need to improve CAR therapy outcomes.

Translating CAR T-cell therapy to AML also requires a careful understanding of characteristics unique to the disease, and the components which drive it. AML is characterized by the presence of heterogeneous blast cells, which are highly aggressive rapidly dividing cells that form the bulk of disease. AML is uniquely challenging to treat due to the role of leukemic stem cells (LSCs) in initiating and maintaining disease [7]. LSCs remain unaffected by chemotherapies targeting rapidly dividing cells due to their quiescent nature. A successful CAR therapy for AML would target two separate antigens to both: (1) combine the bulk targeting of heterogeneous malignant cells with eliminating LSCs that cause relapse and (2) provide coverage of multiple targets to limit single-antigen relapse.

CD33 is a myeloid marker that has been a target of great interest in the treatment of AML due to its specific expression on bulk AML disease and minimal expression on normal cells [1, 8–10]. Patients treated with gentuzumab ozogamicin, an anti-CD33 antibody therapy, relapsed with CD33^+^ AML [8, 11]. Thus, while targeting CD33 eliminates the majority of disease, supplementing with an additional target would help eliminate CD33^−^ leukemic cells or disease-replenishing LSCs. A study of 319 AML patients found that 87.8% of AMLs expressed CD33 [1]. CD123 is also widely present in AML blasts and the same 319 AML patient study found that 9.4% of AMLs express CD123 without CD33. Therefore, targeting CD33 and CD123 together may prevent antigen escape associated with relapse.

CD123 (alpha chain of the interleukin 3 receptor) is an ideal target, as it is overexpressed in AML [12, 13]. Importantly, it displays high expression on CD34^+^CD38^−^ LSCs and is absent from or minimally expressed on normal hematopoietic stem cells (HSCs) [14–16]. CD34^+^CD38^−^ cells are defined as LSCs since they can initiate and maintain the leukemic process in immunodeficient mice. The number of CD34^+^CD38^−^CD123^+^ LSCs is predictive of treatment outcomes for AML patients [7]. Although AML is a heterogeneous disease, the majority of AML samples express either CD33, CD123, or both [1, 13]. Targeting both CD123 and CD33 would, therefore, eliminate AML in the majority of patients.

In our preclinical study, we designed a CD123b-CD33b cCAR expressing discrete anti-CD123 and anti-C33 CAR units to target bulk disease and LSCs simultaneously in AML. Moreover, dual targeting offers more comprehensive ablation and may overcome the pitfalls of single-antigen therapy by preventing relapse due to antigen loss. We showed that CD123b-CD33b cCAR (123b-33bcCAR) T-cells specifically ablated leukemic cells expressing either or both CD123 and CD33 in vitro and in vivo. We also found that the 123b-33bcCAR displayed remarkable efficacy in eliminating both LSCs and leukemia blasts in patient samples. As a safety-switch to protect against the high potency of our cCAR, we developed a strategy that rapidly eliminated all residual cCARs in leukemia mouse models. Together, this study supports the development of 123b-33bcCAR as a promising immunotherapy for AML.

## Materials And Methods

### Construction of xenogenic mouse models

Four mice models were used to analyze anti-leukemia effect and compound antigen targeting: (1) MOLM13 AML tumor model *N* = 4, (2) U937 AML tumor model *N* = 4, (3) Jurkatxp123 single-antigen model *N* = 2, and (4) Jurkatxp33 single-antigen model *N* = 2. Male 12-week-old NSG mice (NOD.Cg-Prkdcsid Il2rgtm1Wjl/SzJ) were purchased from the Jackson Laboratory (Bar Harbor, ME) and used under a Stony Brook University IACUC-approved protocol. NSG mice were given the standard preconditioning sublethal (2.0 Gy) dose of irradiation and then intravenously injected on Day 0 with (1) 1.0 × 10^6^ MOLM13 cells per mouse, (2) 1.0 × 10^6^ U937 cells, (3) 1.0 × 10^6^ Jurkatxp123 cells, or (4) 1.0 × 10^6^ Jurkatxp33 cells per mouse. Three days (Day 3) following tumor cell injection, mice were intravenously injected with one dose of 10 × 10^6^ 123b-33bcCAR T-cells or control T-cells.

Note: For all mice studies, one dose is defined as total cells injected; actual dose of cCAR T-cells is lower due to efficiencies of gene transfer ~25%. Luciferin injection and IVIS imaging were accomplished as previously described [17–20].

Additional methods (including in vitro assays and flow cytometry methods) are described in supplementary materials. The flow cytometry antibodies used are listed in Supplementary Table 1.

## Results

### Generation of CD123-CD33 cCAR (123b-33bcCAR) T-cells

**Fig. 1:**
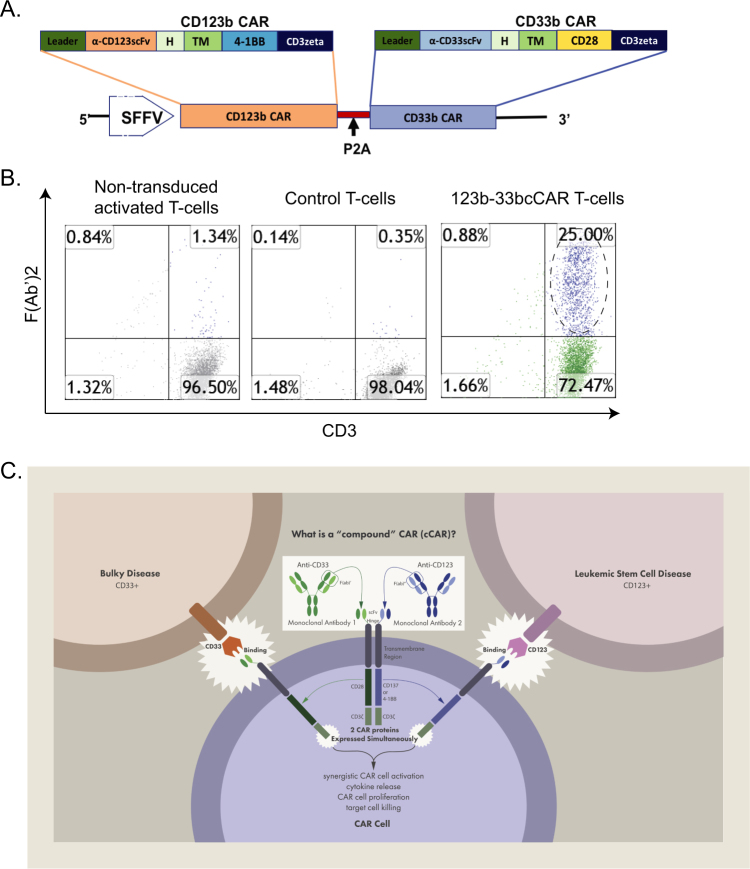
123b-33bcCAR Construct and Expression. **a** Two discrete CAR units: an anti-CD123b CAR comprised of: a CD8-derived hinge (H) and transmembrane (TM) regions, and either 4-1BB or CD28 co-activation domains linked to the CD3ζ signaling domain is fused to a complete anti-CD33b CAR by a self-cleaving P2A peptide. A strong spleen focus forming virus promoter (SFFV) and a CD8 leader sequence were used for efficient expression of the 123b-33bcCAR molecule on the T-cell surface. **b** Expression was measured by FACS against control T-cells. **c** 123b-33bcCAR T-cell dual-pronged approach schematic

The 123b-33bcCAR T-cells were generated by transduction of primary peripheral blood T-cells with the lentiviral construct depicted in Fig. 1a. Flow cytometry analysis showed that ~25% of T-cells expressed the CD123 or CD33 CAR F(Ab′)2 fragment after transduction (Fig. 1b). This 123b-33bcCAR was designed to deplete AML cells by targeting both bulky disease and the CD34^+^CD38^−^CD123^+^ leukemic population to eliminate active disease and prevent leukemia relapses (Fig. 1c).

### 123b-33bcCAR T-cells effectively lyse acute myeloid leukemia cell lines

**Fig. 2:**
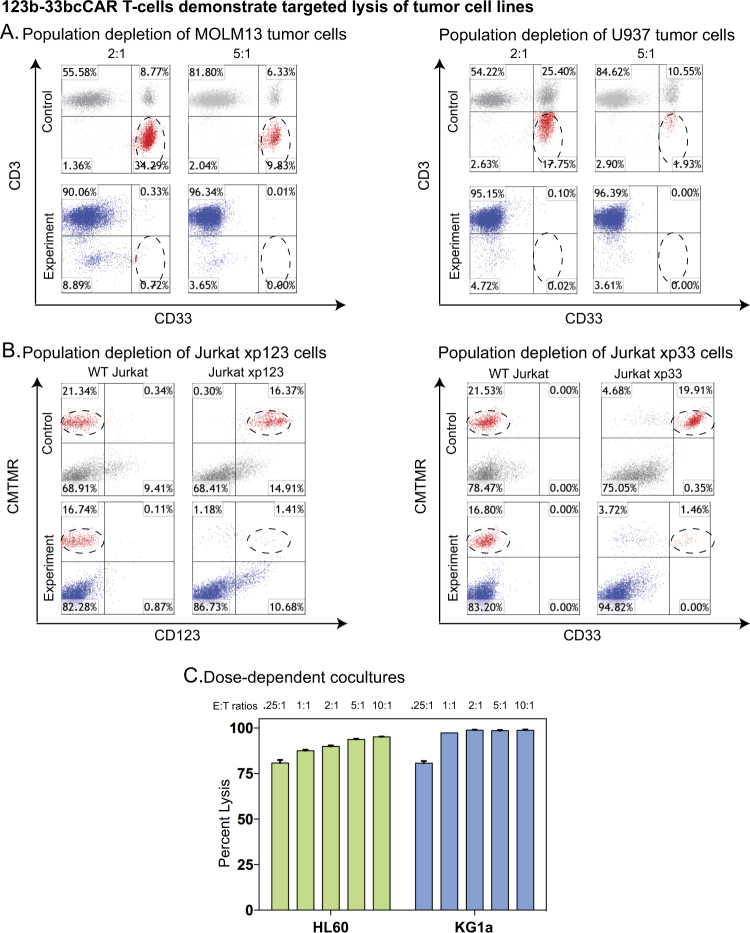
123b-33bcCAR T-cells demonstrate targeted lysis of tumor cells lines. All target populations are encircled. **a** Flow cytometry analysis of control T-cells and 123b-33bcCAR T-cells against MOLM13 (CD123^+^CD33^+^) or U937 (CD123^-^CD33 + ) tumor target cells at 2:1 and 5:1 E:T ratios. **b** Flow cytometry analysis of control T-cells and 123b-33bcCAR T-cells against wild-type (WT) Jurkat tumor cells and Jurkat cells expressing either CD123 (Jurkatxp123) or CD33 (Jurkatxp33) at a 2:1 E:T ratio. **c** Dose-dependent cultures performed with HL60 (CD123^dim^CD33^+^) and KG1a (CD123^+^CD33^+^) cells display high cCAR killing efficiency at E:T ratios ranging from 0.25:1 to 10:1

To assess the cytotoxicity of 123b-33bcCAR T-cells, we conducted co-culture assays against AML cell lines MOLM13 (CD123^+^CD33^+^) and U937 (CD123^−^CD33^+^). FACS analysis of 123b-33bcCAR cytotoxicity in 24 h co-cultures revealed virtually complete lysis (>90%) of MOLM13 and U937 tumor cells at E (effector):T(target) ratios of 2:1 and 5:1 (Fig. 2a, Supplementary Fig. S4A).

We further evaluated the dose-dependent cytotoxicity of the 123b-33bcCAR by progressively decreasing the E:T ratio against two other AML cell lines: KG1a (CD123^+^CD33^+^) and HL60 (CD123^dim^CD33^+^). Using E:T ratios of: 0.25:1, 0.5:1, 1:1, 2:1, 5:1, and 10:1, 123b-33bcCAR displayed robust lysis even at the 0.25:1 ratio (Fig. 2c). Leukemic cell phenotypes are included in Supplementary Fig. S1.

### 123b-33bcCAR T-cells’ discrete receptor units independently lyse target cells in an antigen-specific manner

To further confirm our cCAR’s independent antigen targeting ability, T-ALL Jurkat cell lines were overexpressed with either CD123 (Jurkatxp123) or CD33 (Jurkatxp33) and independent antigen expression was confirmed (Supplementary Fig. S1). To determine targeting functionality, 123b-33bcCAR T-cells were co-cultured against these cells in addition to wild-type Jurkat cells (Fig. 2b). We found that the 123b-33bcCAR T-cells specifically and potently ablated (>90% lysis) Jurkatxp123 and Jurkatxp33 cells when compared to wild-type Jurkat cells expressing neither antigen (Supplementary Fig. S4B). Overall, 123b-33bcCAR T-cells displayed effective bulk cytotoxicity, ablating cell populations expressing varying combinations of targets, and cells expressing only each individual antigen (Fig. 2).

### 123b-33bcCAR T-cells effectively lyse primary leukemia tumor cells

**Fig. 3:**
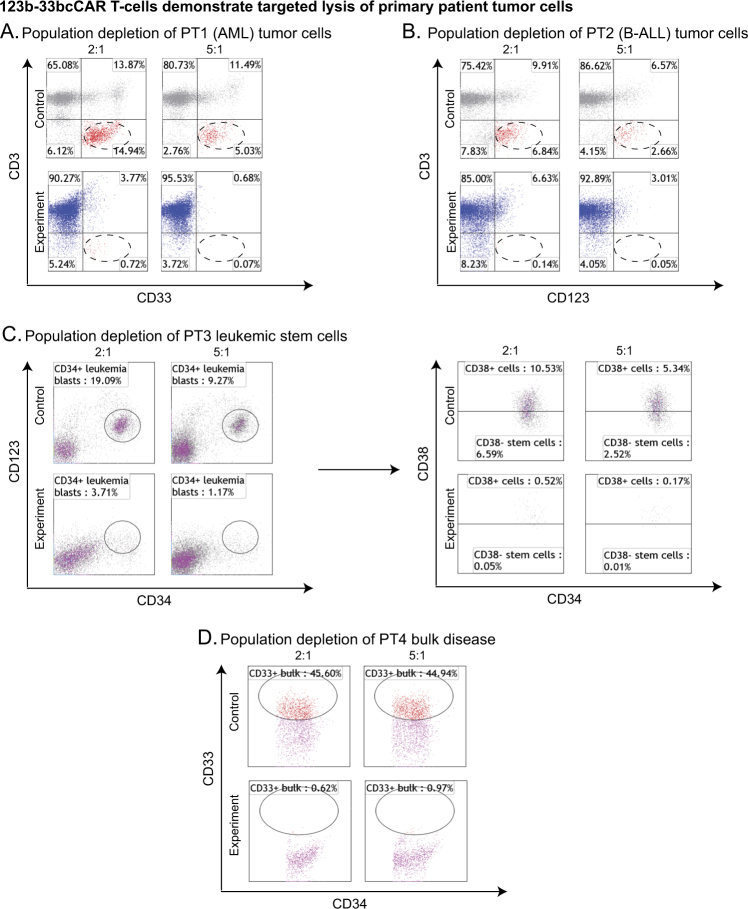
123b-33bcCAR T-cells demonstrate targeted lysis of primary patient tumor cells. All target populations encircled. **a** Flow cytometry analysis of control T-cells and 123b-33bcCAR T-cells against PT1 tumor target cells at 2:1 and 5:1 E:T ratios. **b** Flow cytometry analysis of control T-cells and 123b-33bcCAR T-cells against PT2 tumor target cells at 2:1 and 5:1 E:T ratios. **c** Flow cytometry analysis of control T-cells and 123b-33bcCAR T-cells against PT3 tumor target cells at 2:1 and 5:1 E:T ratios. The target cell population (CD123^+^CD34^+^) is further broken down by CD38 expression to display LSC (CD123^+^CD34^+^CD38^-^) elimination. **d** Flow cytometry analysis of control T-cells and 123b-33bcCAR T-cells against PT4 tumor target cells (CD33^+^ bulk disease) at 2:1 and 5:1 E:T ratios

We next established the anti-tumor properties of the 123b-33bcCAR against four primary patient leukemia samples, including two CD123^+^CD33^+^ AML and two CD123^+^ B-ALL samples (PT1:AML, PT2:B-ALL, PT3:AML, and PT4:B-ALL) (Fig. 3, Supplementary Fig. S4C). Compared to the previous anti-tumor cytotoxicity against AML cell lines (Fig. 2), 123b-33bcCAR T-cells showed similarly potent anti-leukemic activity against all patient samples, with >80% tumor lysis at the 2:1 ratio and >98% tumor lysis at the 5:1 ratio.

We also examined the ability of our 123b-33bcCAR to eliminate specific cell populations including LSCs (CD123^+^CD34^+^CD38^−^) in the PT3 sample and myeloid leukemia bulk disease (CD34^variable^CD33^+^) in the PT4 sample (Fig. 3c, d). We found that 123b-33bcCAR T-cells successfully ablated both LSCs and bulk disease cells. Patient sample phenotypes are included in Supplementary Fig. S2.

### 123b-33bcCAR T-cells exhibit profound anti-tumor activity in vivo

**Fig. 4:**
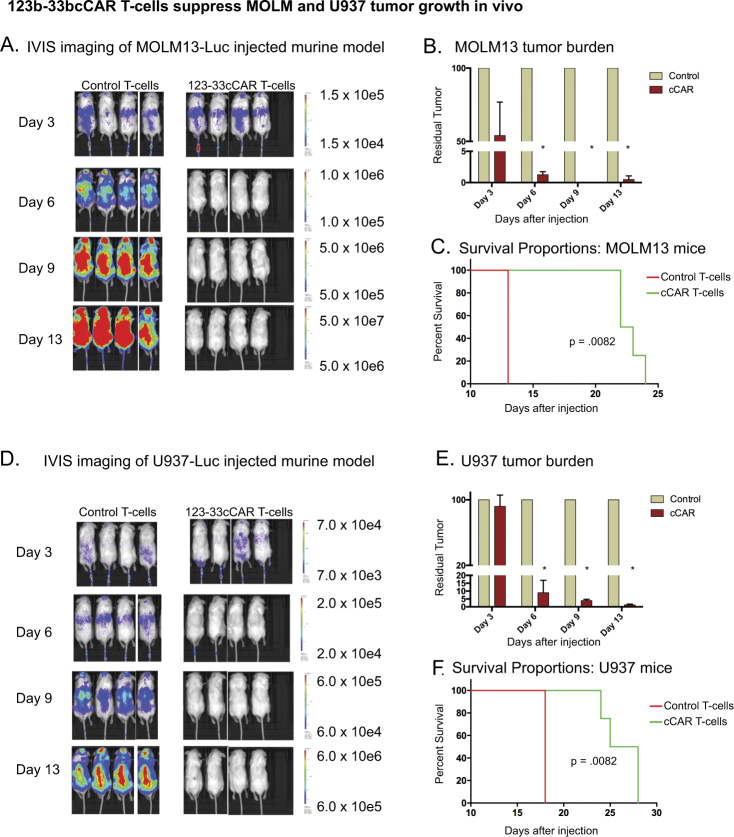
123b-33bcCAR T-cells demonstrate a profound anti-leukemic effect against MOLM13 and U937 cell lines in two in vivo xenograft mouse models. **a** Mouse model using MOLM13 cells to induce measurable tumor formation. Mice treated with either 123b-33bcCAR T-cells or control T-cells. Tumor burden visualized on days 3, 6, 9, and 13 (left panel) with **b** graphical representation on the right. **c** Log-rank Mantel–Cox test shows significance for improved experimental group survival (*P* = 0.0082). **d** Mouse model using U937 cells to induce measurable tumor formation. Mice treated with either 123b-33bcCAR T-cells or control T-cells. Tumor burden visualized on days 3, 6, 9, and 13 (left panel) with **e** graphical representation on the right. **f** Log-rank mantel-cox test shows significance for improved experimental group survival (*P* = 0.0082)

In order to evaluate the in vivo anti-tumor activity of 123b-33bcCAR T-cells, we developed two mouse models with either luciferase-expressing MOLM13 (CD123^+^CD33^+^) or U937 (CD123^−^CD33^+^) cells to induce fluorescence visible tumor formation (Fig. 4a, d). The 123b-33bcCAR cells significantly reduced tumor burden and prolonged survival in MOLM13^−^injected and U937-injected mice. Mice were given a single dose of 123b-33bcCAR T-cells or control T-cells cells and tumor burden assayed by IVIS imaging. There was a significant difference (*P* < 0.0001) in IVIS measurement of tumor burden between the control group and the 123b-33bcCAR treatment group from Day 6 onwards in both xenograft models (Fig. 4b, e). Mice injected with 123b-33bcCAR T-cells had ~92-99% less tumor burden than control mice by Day 13. Moreover, cCAR-treated mice also had significantly more favorable survival outcomes (*P*-value = 0.0082 for Mantel–Cox in both groups) (Fig. 4c, f).

We also evaluated tumor cell and CAR T-cell persistency at the time of sacrifice. Peripheral blood was collected and analyzed via flow cytometry for the presence of transplanted tumor (MOLM13 or U937 cells) and T-cells (cCAR or control). While control-treated mice showed significant residual tumor populations (~75–87%) in the peripheral blood, 123b-33bcCAR treated mice showed virtual depletion of all tumor cells (Supplementary Fig. S3). In addition, 123b-33bcCAR treated mice showed significant T-cell expansion and persistence, whereby virtually all the human cells found in the peripheral blood were cCAR T-cells. These findings correlated with observed robust anti-leukemia activity and supported overall significantly improved survival.

### 123b-33bcCAR T-cells display discrete antigen targeting ability in vivo

**Fig. 5:**
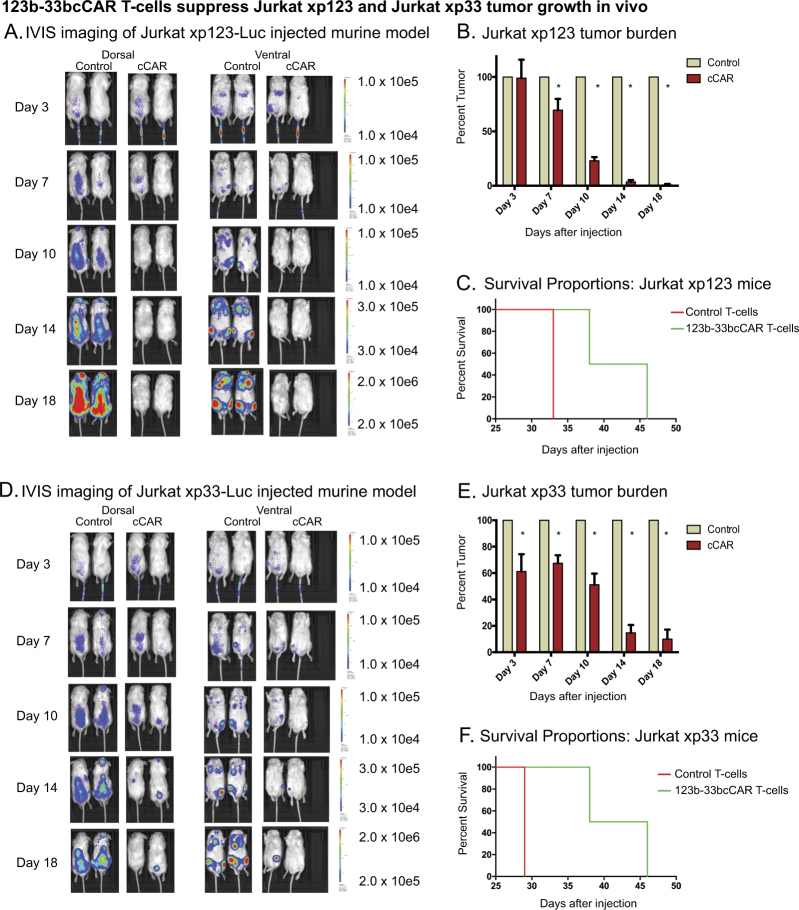
123b-33bcCAR T-cells display discrete antigen targeting in two in vivo xenograft mouse models. **a** Mouse model using Jurkatxp123 cells to induce measurable tumor formation. Mice treated with either 123b-33bcCAR T-cells or control T-cells. Tumor burden visualized on days 3, 7, 10, 14, and 18 (left panel) with **b** graphical representation on the right. **c** Kaplan–Meier survival curve represents survival outcomes. **d** Mouse model using Jurkatxp33 cells to induce measurable tumor formation. Mice treated with either 123b-33bcCAR T-cells or control T-cells. Tumor burden visualized on days 3, 7, 10, 14, and 18 (left panel) with **e** graphical representation on the right. **f**) Kaplan–Meier survival curve represents survival outcomes

To assay specific CD123^+^ or CD33^+^ cell depletion and verify compound scFv efficacy, two additional xenograft mouse models of artificial tumor cells independently expressing each antigen (Jurkatxp123 or Jurkatxp33) were generated (Fig. 5). Both groups were treated with 123b-33bcCAR and control T-cells to test 123b-33bcCAR’s independent antigen targeting ability. There was a significant difference (*P* < 0.002) in IVIS measurement of tumor burden between the control group and the 123b-33bcCAR treatment group from day 7 onwards for the Jurkatxp123 mice and as early as day 3 for the Jurkatxp33 mice (Fig. 5b, e). By day 18, tumor cells were virtually absent (<1% tumor burden) in 123b-33bcCAR-treated Jurkatxp123 mice, and Jurkatxp33 mice had only 10% residual tumor.

### 123b-33bcCAR T-cells can be rapidly depleted by alemtuzumab in vivo

The development of safety methods to eliminate 123b-33bcCAR T-cells from AML patients after tumor depletion may be necessary in emergencies due to unexpected side effects of cCAR therapy. T-cells express CD52 on the cell surface and a CD52-specific antibody, CAMPATH (alemtuzumab), can eliminate CD52^+^ cells from circulation.

**Fig. 6:**
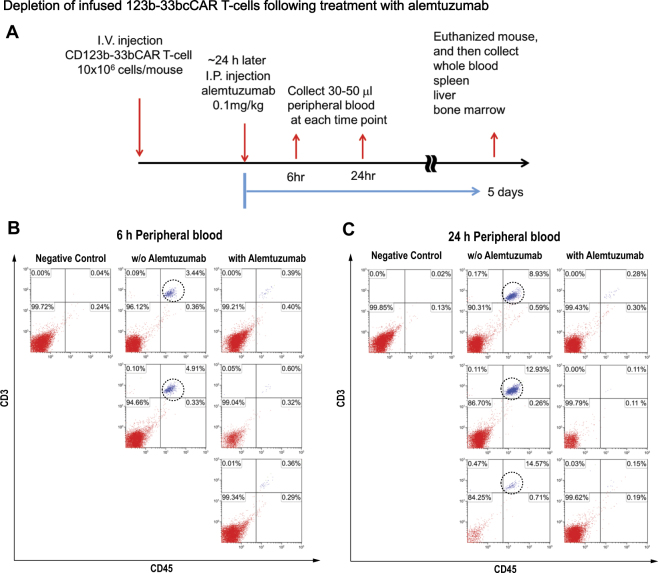
Depletion of infused 123b-33bcCAR T-cells following treatment with alemtuzumab. **a** Experimental schema to evaluate the effect of alemtuzumab administration after 123b-33bcCAR T-cells infusion into NGS mice. **b** Representation of 123b-33bcCAR T-cells persistence in peripheral blood 6 h later. Presence of 123b-33bcCAR T-cells was detected by flow cytometry. **c** Representation of persistence of infused 123b-33bcCAR T-cells in peripheral blood 24 h later. Presence of 123b-33bcCAR T-cells was detected by flow cytometry

To evaluate the feasibility of an alemtuzumab-mediated depletion strategy as a natural safety-switch for 123b-33bcCAR T-cells, we established a mouse model with a systemic injection of 123b-33bcCAR T-cells. Mice were treated with alemtuzumab and the tissues were analyzed as described in the workflow in Fig. 6a. After 6 h of alemtuzumab administration, flow cytometry analysis confirmed ~90% depletion of 123b-33bcCAR T-cells in the circulating peripheral blood (Fig. 6b, Supplementary Fig. S5A), and the depletion rate increased to >98% 48 h post-alemtuzumab (Fig. 6c, Supplementary Fig. S5A). Furthermore, 5 days post-alemtuzumab, the 123b-33bCAR T-cells were virtually eradicated from the peripheral blood, spleen, liver, and bone marrow (Supplementary Fig. S5B). Alemtuzumab, therefore, rapidly and efficiently eliminated both peripheral blood cCAR T-cells as well as tissue-infiltrating cCAR T-cells. These findings support the use of alemtuzumab as a natural safety-switch to deplete 123b-33bcCAR T-cells from circulation.

## Discussion

While initial remission rates of ~90% are commonly seen in CD19CAR-treated B-ALL patients, most relapse within a year. The relapse may arise from incomplete chimeric antigen coverage resulting in antigen escape. A single target for CAR-based treatment may, therefore, not be sufficient to prevent leukemia relapse and more effective CAR T-cell treatments to prevent relapse are urgently needed. Single-CAR treatment of AML poses similar concerns for antigen escape, emphasizing the importance of dual targeting approaches.

CD123 and CD33 are good cCAR targets as virtually all AML cells express one or both of these antigens. We developed a cCAR targeting both CD123 and CD33 antigens simultaneously as a strategy for more comprehensive disease ablation. Dual targeting of AML by a combined CD123^−^CD33^−^directed therapy may overcome the pitfalls of single-antigen therapy by preventing relapse due to antigen loss. While loss of a single antigen under antigen-specific selection pressure is possible, loss of two antigens simultaneously is much more unlikely.

One important prognostic indicator of AML is minimal residual disease, defined as small numbers of leukemic cells which remain after treatment. This population is predominantly comprised of LSCs and is able to escape conventional chemotherapy, resulting in relapse [7]. Purified CD34 + CD38^−^ leukemic cells are essentially LSCs that overexpress CD123. While current therapies aim to de-bulk the disease by logs of depletion of these cells, they fail to eliminate LSCs, thus allowing the regeneration of disease and blasts. A more successful strategy is to simultaneously target the roots of the disease (CD123 + CD34 + CD38- LSCs) and bulk of the disease (CD33 + blasts).

A variety of approaches have been used to target either CD123 or CD33 in AML patients, including antibody-drug conjugates and T-cell recruiting antibody constructs [21]. These approaches have met limited success with high rates of relapse, which may be due to an inability to simultaneously target bulk disease and LSCs. Several clinical trials have employed CARs to individually target either CD123 (NCT02159495, NCT03190278) or CD33 (NCT01864902; NTC0186902; NCT02944162; NCT03126864); although sparse preliminary data supports limited success. In a small clinical trial using CD33 CAR T-cells to treat one patient with refractory AML, CD33^+^ blast cells were decreased at two weeks, but had returned to high numbers by week 9 after treatment, accompanied by undesirable side effects [22]. The same group also treated a single patient with anti-CD123 CAR T-cells, and reported no clear anti-leukemic effect. Although not enough data from these trials have been published to draw conclusions, these early results suggest that single-antigen targeting might not be sufficient to stop AML development and disease progression.

To offer more potent and comprehensive coverage to eliminate both LSCs and bulk disease and to minimize antigen escape, we propose a cCAR therapy combining both the CD123 and CD33 targets. The FDA has granted this 123b-33bcCAR orphan drug designation for AML treatment (#17-6031). To study the efficacy of the 123b-33b compound CAR (123b-33bcCAR), we identified three criteria for its feasibility for the treatment of AML. First, its basic cytotoxic functionality to ablate AML cell lines and patient tumor samples. Second, the ability of each discrete cCAR unit to independently target its antigen and eliminate a target expressing only one antigen or both antigens, both in vitro and in vivo. Third, our ability to rapidly and completely terminate the cCAR in vivo.

We first showed that the 123b-33bcCAR had impressive cytotoxic activity against leukemic cell lines and patient tumors, reaching a nearly 100% killing rate. The cCAR targeted and responded to a variety of primary leukemia samples and efficiently ablated CD123^+^ LSCs and CD33^+^ AML bulk cells in these samples. We further demonstrated the remarkable killing capacity of the cCAR through dose-dependent co-cultures reaching effector:target (E:T) cell ratios as low as 0.25:1. Low-dose co-cultures with the 123b-33bcCAR T-cells were comparable to higher E:T ratios, suggesting that the cCAR technology is incredibly powerful.

We next validated the ability of compound therapy to target multiple antigens by independently testing each target (CD123^+^CD33^−^ or CD123^-^CD33^+^). We showed that the 123b-33bcCAR was able to ablate both the CD123-expressing Jurkatxp123 cells and the CD33-expressing Jurkatxp33 cells independently both in co-culture assays and in mouse models. Mice treated with 123b-33bcCAR showed trends of improved survival as compared to control-treated mice. We also showed the 123b-33bcCAR promoted sustained in vivo activity against the MOLM13 and U937 cell lines, as well as superior murine survival in both models. The eventual leukemia relapse in the mice may have been due to the limited ability of human CAR T-cells to effectively penetrate all murine reservoirs. The presence of murine reservoirs that harbor leukemia and allow relapse have been documented by other groups [17, 23–25]. Moreover, mice bearing xenografts of human malignancies do not recapitulate the human microenvironment, which allows for 1000- to 10,000-fold CAR T-cell expansion [26]. Importantly, all cCAR-treated mouse hematological tissues were largely free of tumor with large populations of expanded 123b-33bcCAR T-cells at these endpoints, suggesting that each unit is fully functional and independently sufficient.

A major concern in translating our 123b-33bcCAR to the clinic is the potential for “on-target off-tumor” effects. To rapidly eliminate our powerful cCAR T-cells in the clinic, we employed an alemtuzumab-mediated depletion strategy. Within 6 h of low-dose (0.1 mg/kg) alemtuzumab safety-switch administration, 90% of cCAR T-cells were eliminated from murine blood and tissues, with almost no cells remaining at 24 h. Another group showed that 4–6 weeks of cCAR therapy was sufficient to eliminate leukemic disease in vivo, and subsequent alemtuzumab administration did not hamper CAR therapy [27].

The 123b-33bcCAR offers a great opportunity in the clinical setting of AML treatment. Since many AML patients relapse within a short time of induction and pre-transplant, the successful eradication of AML blasts and LSCs by 123b-33bcCAR T-cells may provide a better window of opportunity to treat these patients with allogeneic stem cell transplant. As a bridge to transplant, the cCAR would be administered at a specified dose and the disease status would be monitored closely for 4–6 weeks. Preparation of allogeneic transplant would begin once no evidence of disease remains. The patient would receive the conditioning regimen, followed by a low dose of alemtuzumab, and finally a stem cell transplant.

There is a dire need for a new AML treatment. Current clinical trials targeting various antigens via CAR T-cells hold great promise, but there is limited published data. Preliminary results from these trials suggest that single-antigen approaches may not be enough. As evidenced by the current treatment regimens, a means of eliminating the LSCs comprising minimal residual disease while also ablating the bulk leukemia, effectively uprooting the AML tree, is essential for long-term results. Our work supports a compound CAR as a novel and powerful means of advancing current AML therapies.

## Electronic supplementary material

Revised Additional methods and materials_clean

Supplementary Figure Legends

Leukemia cell line phenotypes

Patient sample phenotypes

123b-33bcCAR T-cells efficiently eliminate tumor and display high persistency

Percent Lysis Summary

Alemtuzumab bar graph

Reagents table
