# Supplementary material for: Compound CAR T-cells as a double-pronged approach for treating acute myeloid leukemia
Source: Leukemia. 2018 Feb 25;32(6):1317–26. doi: 10.1038/s41375-018-0075-3 (PMC5990523; doi:10.1038/s41375-018-0075-3)
Supplement: Supplementary file 1 — Revised Additional methods and materials_clean [file 41375_2018_75_MOESM1_ESM.docx]

**ADDITIONAL METHODS AND MATERIALS**

**Primary tumor cells and cell lines**

Human primary leukemia samples were obtained from residual samples following a protocol approved by the Institutional Review Board of Stony Brook University. MOLM-13 cell line was obtained from AddexBio (San Diego, CA, USA). U937 and Jurkat cell lines were obtained from ATCC (Manassas, VA, USA). CD123 and CD33 expressing Jurkat cells (Jurkat xp123, Jurkat xp33) were generated by CD123b and CD33b cDNA transduction, respectively, and stably maintained by puromycin selection (1.0 ug/ul). MOLM13, U937, and Jurkat xp cell lines were cultured in RPMI, 10% FBS, 1% Pen/Strep (Gibco). T-cells and patient samples were cultured in filtered T cell media, defined as 50% AIM V, 40% RPMI 1640 and 10%FBS, with 1% Pen/Strep (all Gibco, Waltham, MA, USA) and supplemented with IL-2 (300 IU/mL; Peprotech, Rocky Hill, NJ, USA).

**CAR construct design, lentiviral transduction, and detection**

The CD123b-CD33b-41BB/CD28-2G (abbv. 123b-33bcCAR) in a pRSC vector consists of two independent, discrete CAR units driven by a SFFV promoter fused together by a self-cleaving P2A peptide. Each CAR unit is comprised of an intracellular tandem signaling domain made up of either 4-1BB (CD123) or CD28 (CD33) and CD3ζ domains and upon export to cell surface, are expressed in roughly equal ratios based the P2A cleavage efficiency^1, 5^. A 123b-33bcCAR T-cell is defined as having a complete CD123 CAR and a complete CD33 CAR expressed on the same cell, with independent targeting and signaling. Viral supernatant containing the 123b-33bcCAR was produced by 293T-cells co-transfected with pMD2G and pSPAX viral packaging plasmids containing either pRSC.SFFV.cCAR.2G or control vector control using Lipofectamine 2000 (Life Technologies, Carlsbad, CA) as per manufacturer’s protocol. T-cells were obtained from four healthy donors, as T-cell origin does not significantly impact CAR killing ability. CAR killing ability depends on transduction efficacy, with higher transduction rates resulting in higher lysis ability. T-cells were activated and transduced as previously described, in which T-cells are incubated in BSA-blocked 12 well plates with lentiviral supernatant containing the 123b-33bcCAR or vector control construct overnight ^2,3,5^.

CAR T-cells were assayed for expression using flow cytometry for F(ab)’ fragment detection as previously described ^2,3,5^. Flow cytometry was performed using a FACS Calibur instrument (Becton Dickinson, Franklin Lakes, NJ), and results were analyzed using Kaluza software (Beckman Coulter, CA).

**Co-culture assays and gating schemes**

123b-33bcCAR and vector control T-cells were incubated with CD123 and/or CD33 expressing leukemia cell lines: MOLM13 (n=2), U937 (n=2), HL60 (n=2), KG1a (n=2) in addition to primary patient cells two CD123+CD33+ AML and two CD123+ B-ALL samples [PT1:AML (n=2), PT2:B-ALL (n=2), PT3:AML (n=2), and PT4:B-ALL (n=2)]. For antigen specific assays, we used stably transduced CD123 or CD33 expressing Jurkat cells (under puromycin selection) (n=2). As a negative control, 123b-33bcCAR and control T-cells were incubated with CD123 negative and CD33 negative wild-type Jurkat cells (n=2).

Co-cultures were carried out at various E:T (effector to target cell) ratios where marked. After 24 hours, remaining live cells were harvested and stained with a combination of mouse anti-human CD3, anti-CD123, anti-CD33, anti-CD34, or anti-CD38 antibodies for cytotoxicity assays. Target cells were pre-labeled with cytotracker (CMTMR) when necessary for clear target and effector cell population separation. For cell line and primary cell phenotypes refer to Supplementary Figures 1 and 2. All cells were washed with FACS buffer, re-suspended in 2% formalin, and analyzed by flow cytometry and analysis of anti-leukemic activity was done as previously described ^2–5^.

**Safety Switch**

In order to evaluate the safety switch to eliminate cCAR T-cells *in vivo*, a total of 6 NSG mice were intravenously injected with 10 x 10^6^ 123b-33bcCAR T-cells after sublethal (2.0 Gy) irradiation. On the following day, 0.1mg/kg of alemtuzumab (n=3) or PBS (n=3) was administrated via IP injection to each group. After 6h and 24h of alemtuzumab administration, peripheral blood was collected from the mouse tail and the presence of 123b-33bcCAR T-cells was determined by flow cytometry using anti-human CD3-PE (Tonbo) and CD45-PerCp (Tonbo) labeled antibodies. After 5 days of alemtuzumab administration, mice were sacrificed and the whole blood, spleen, liver, and bone marrow cells were harvested and analyzed by flow cytometry to confirm 123b-33bcCAR T cell depletion in the circulation and in the different tissues.

**Mouse model analysis**

Xenogeneic model sample sizes were estimated using 2-sample, 2-sided equality power analysis (90% power and <5% significance) without blinding. Unpaired student t-tests were used to determine significance of tumor size area and light intensity. Survival curves were constructed using the Kaplan-Meier method and statistical analyses of survival was performed using a log-rank (Mantel-Cox) test with P <0.05 considered significant. Statistical analyses were performed using GraphPad Prism 6 software. Variance was determined to be similar between the treatment and control group prior to unpaired student t-tests.

1. Kim, J. H. et al. High Cleavage Efficiency of a 2A Peptide Derived from Porcine Teschovirus-1 in Human Cell Lines, Zebrafish and Mice. PLOS ONE 6, e18556 (2011).

2. Chen, K. H. et al. Preclinical targeting of aggressive T-cell malignancies using anti-CD5 chimeric antigen receptor. Leukemia (2017). doi:10.1038/leu.2017.8

3. Pinz, K. et al. Preclinical targeting of human T-cell malignancies using CD4-specific chimeric antigen receptor (CAR)-engineered T-cells. Leukemia 30, 701–707 (2016).

4. Chen, K. H. et al. Novel anti-CD3 chimeric antigen receptor targeting of aggressive T cell malignancies. Oncotarget 7, 56219–56232 (2016).

5. Chen, K. H. et al. A compound chimeric antigen receptor strategy for targeting multiple myeloma. Leukemia. Epub ahead of print (2017). doi: 10.1038/leu.2017.302.
