# Supplementary material for: Compound CAR T-cells as a double-pronged approach for treating acute myeloid leukemia
Source: Leukemia. 2018 Feb 25;32(6):1317–26. doi: 10.1038/s41375-018-0075-3 (PMC5990523; doi:10.1038/s41375-018-0075-3)
Supplement: Supplementary file 2 — Supplementary Figure Legends [file 41375_2018_75_MOESM2_ESM.docx]

**SUPPLEMENTARY FIGURE LEGENDS**

**Figure S1. Leukemia cell line phenotypes. (A)** CD123 and CD33 marker profiles of leukemia cell lines via flow cytometry analysis. **(B)** CD123 and CD33 marker profiles of artificial cell lines via flow cytometry analysis. Histogram display compares WT Jurkat cells to Jurkat xp33 and Jurkat xp123 cells artificially induced to express each marker.

**Figure S2. Patient sample phenotypes.** CD123 and CD33 marker profiles of four patient cell lines via flow cytometry analysis.

**Figure S3. 123b-33bcCAR T-cells efficiently eliminate tumor and display high persistency.** Peripheral blood of MOLM13 and U937 mice tumor models. Flow cytometry allowed visualization of CD45^+^CD3^+^ T-cells and CD45^+^CD33^+^ tumor cells. **(A)** Flow cytometry analysis of blood of three MOLM13 tumor-injected mice treated with either control T-cells or 123b-33bcCAR T-cells. Tumor cells are depicted in red. **(B)** Flow cytometry analysis of blood from three U937 tumor-injected mice treated with either control T-cells or 123b-33bcCAR T-cells. Tumor cells are depicted in red.

­­

**Figure S4. 123b-33bcCAR co-culture percent lysis summaries.**

**(A)** Percent lysis summary of 123b-33bcCAR T-cells against MOLM13 and U937 leukemia cell lines at both 2:1 and 5:1 E:T ratios. **(B)** Percent lysis summary of 123b-33bcCAR T-cells against wild-type Jurkat, and Jurkat xp123 and Jurkat xp33 artificial cell lines. **(C)** Percent lysis summary of 123b-33bcCAR T-cells against all four patient samples at both 2:1 and 5:1 E:T ratios.

**Figure S5. Alemtuzumab depletion of 123b-33bcCAR *in vivo*.**

**(A)** Depletion of CD123b-33b-CAR T-cells in peripheral blood 6h and 24h after alemtuzumab injection. **(B)** Depletion of 123b-33bcCAR T-cells in mouse whole blood, spleen, liver and bone marrow 5d after alemtuzumab infusion.
