# Supplementary figures and images for: Compound CAR T-cells as a double-pronged approach for treating acute myeloid leukemia
Source: Leukemia. 2018 Feb 25;32(6):1317–26. doi: 10.1038/s41375-018-0075-3 (PMC5990523; doi:10.1038/s41375-018-0075-3)

Figure S1

A. Leukemia cell lines

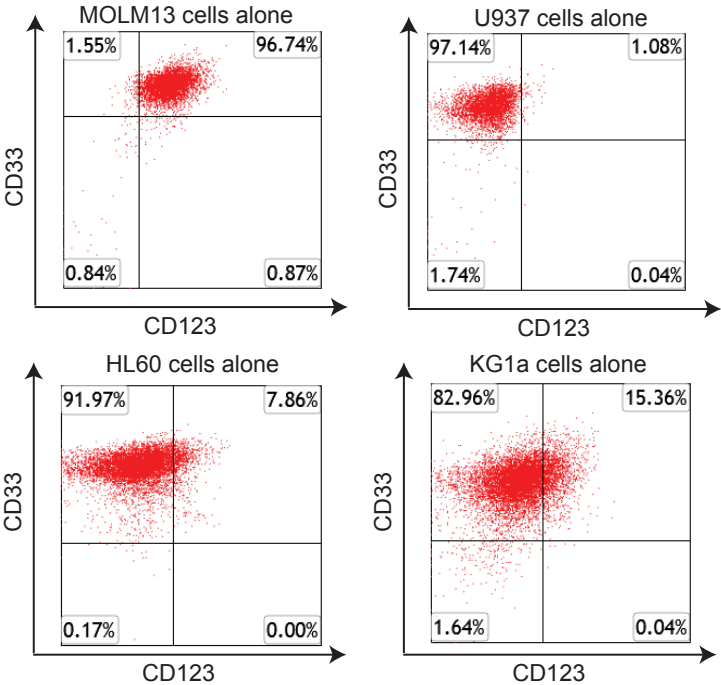

B. Artificial cell lines

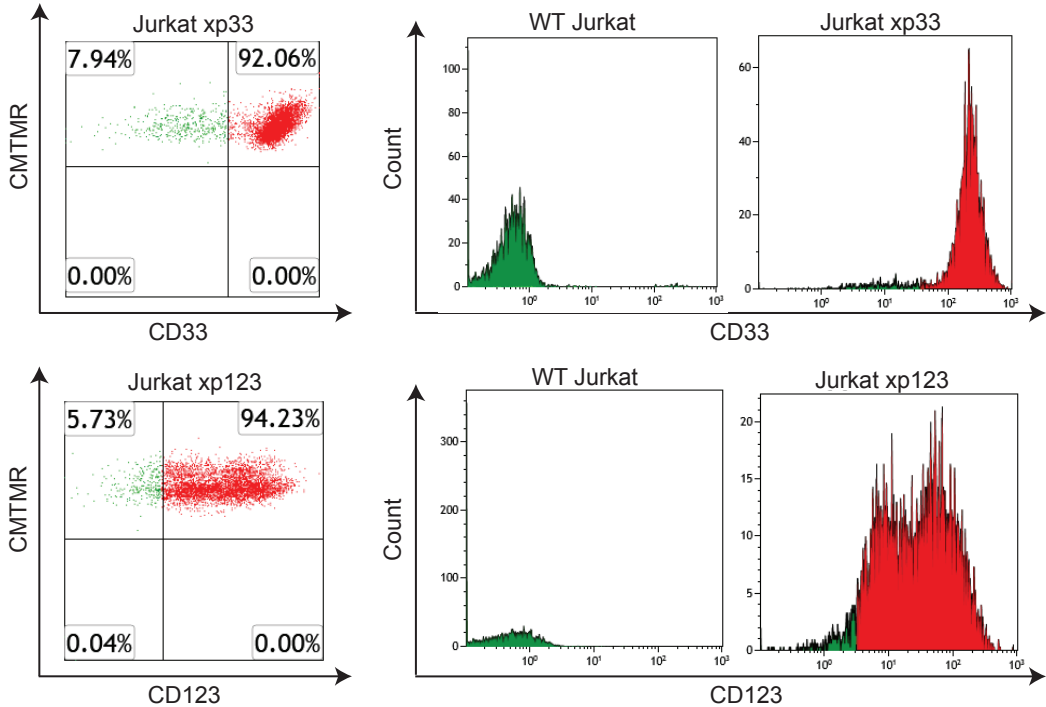

Supplement: Supplementary file 3 — Leukemia cell line phenotypes [file 41375_2018_75_MOESM3_ESM.pdf]

Figure S2

Patient Sample Phenotypes

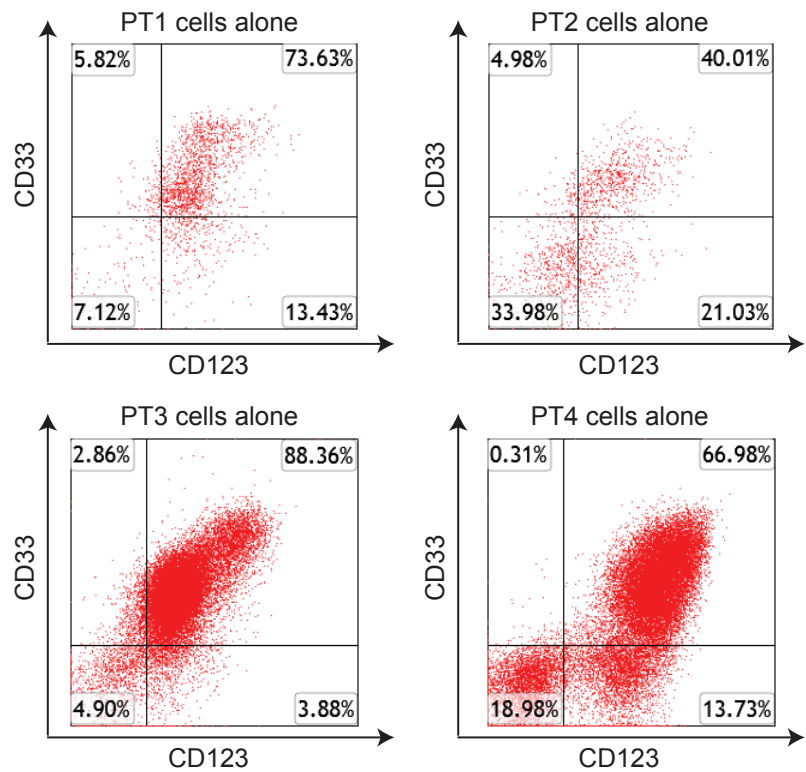

Supplement: Supplementary file 4 — Patient sample phenotypes [file 41375_2018_75_MOESM4_ESM.pdf]

**Figure S3**

Peripheral blood tumor clearance and cCAR persistency

A. MOLM13 mice

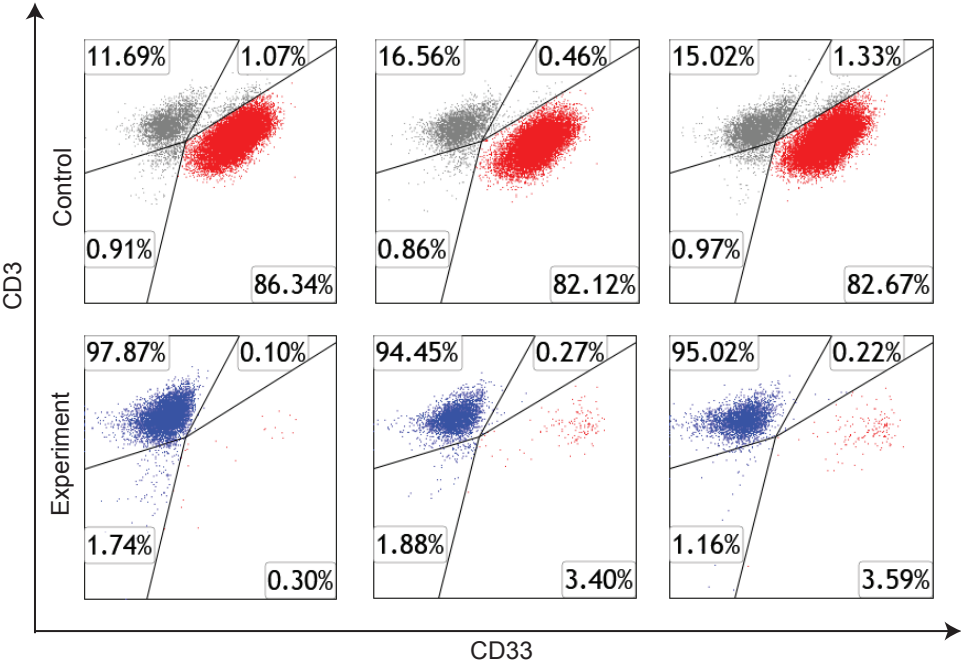

B. U937 mice

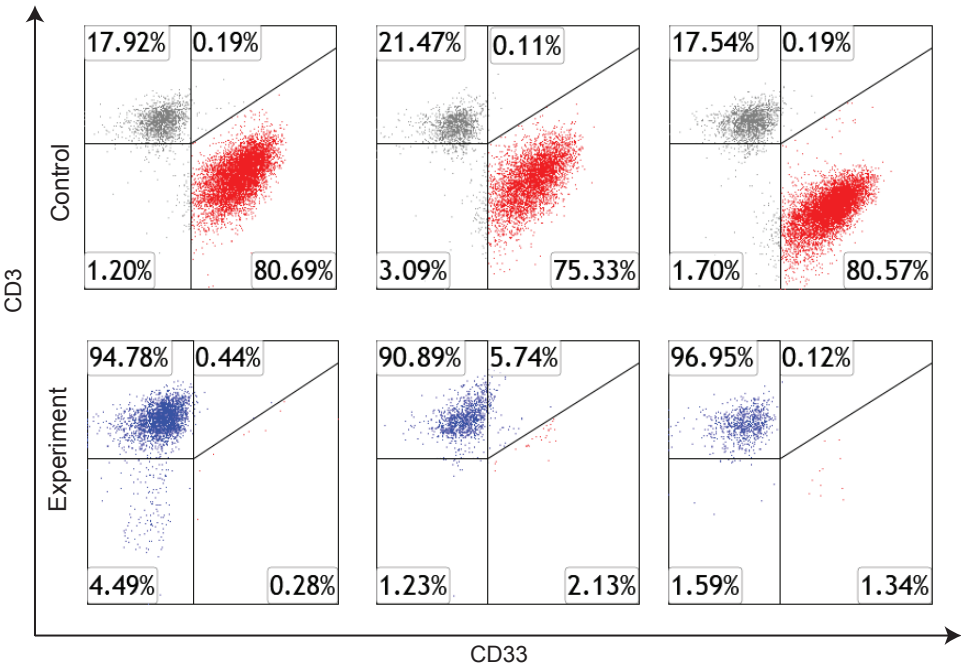

Supplement: Supplementary file 5 — 123b-33bcCAR T-cells efficiently eliminate tumor and display high persistency [file 41375_2018_75_MOESM5_ESM.pdf]
