# Supplementary material for: Compound CAR T-cells as a double-pronged approach for treating acute myeloid leukemia
Source: Leukemia. 2018 Feb 25;32(6):1317–26. doi: 10.1038/s41375-018-0075-3 (PMC5990523; doi:10.1038/s41375-018-0075-3)

Figure S5

Alemtuzumab depletion of 123b-33bcCAR in vivo

A. 123b-33bcCAR depletion in blood

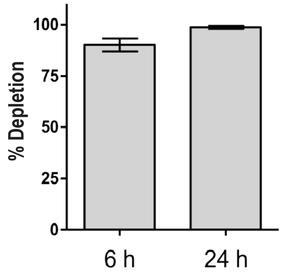

B. 123b-33bcCAR depletion in various tissues (5d)

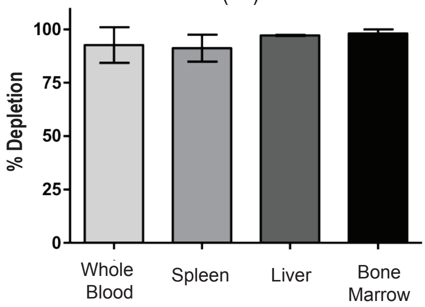

Supplement: Supplementary file 7 — Alemtuzumab bar graph [file 41375_2018_75_MOESM7_ESM.pdf]
