# Supplementary material for: Compound CAR T-cells as a double-pronged approach for treating acute myeloid leukemia
Source: Leukemia. 2018 Feb 25;32(6):1317–26. doi: 10.1038/s41375-018-0075-3 (PMC5990523; doi:10.1038/s41375-018-0075-3)
Supplement: Supplementary file 8 — Reagents table [file 41375_2018_75_MOESM8_ESM.docx]

| **NAME** | **VENDOR** | **HOST SPECIES** | **CATALOG #** | **CLONE #** | **DILUTION** | **USE** |
| --- | --- | --- | --- | --- | --- | --- |
| Goat anti-mouse F(AB’)^2^, biotin | Jackson | Goat | 115-066-072 | n/a | 1:250 | F(Ab')2 detection/Flow cytometry |
| R-Phycoerythrin Streptavidin | Jackson | n/a | 016-110-084 | n/a | 1:250 | Secondary for F(Ab')2 detection/Flow cytometry |
| Anti-human CD3-PerCP | Tonbo Biosciences | Mouse | 65-0037-T025 | OKT3 | 1:50 | Flow cytometry |
| Anti-human CD3-PE | Tonbo Biosciences | Mouse | 50-0037-T-100 | OKT3 | 1:50 | Flow cytometry |
| Anti-human CD123-PerCP | BD Biosciences | Mouse | 558714 | 7G3 | 1:50 | Flow cytometry |
| Anti-human CD33-APC | Biolegend | Mouse | 366606 | P67.6 | 1:50 | Flow cytometry |
| Anti-human CD34-PeCy7 | BD Biosciences | Mouse | 560710 | 581 | 1:50 | Flow cytometry |
| Anti-human CD38 Alexa Fluor F450 | Tonbo Biosciences | Mouse | 75-0389-T`00 | HIT2 | 1:50 | Flow cytometry |
| Anti-human CD45-Krome Orange | Beckman Coulter | Mouse | A96416 | J.33 | 1:50 | Flow cytometry |
| CMTMR | Life Technologies | n/a | C2927 | n/a | 5 μM | Flow cytometry |

**Supplementary Table 1: Flow cytometry antibodies**
